# Label-Free Mass Spectrometry-Based Quantitative Proteomics Analysis of Serum Proteins During Early Pregnancy in Jennies (*Equus asinus*)

**DOI:** 10.3389/fvets.2020.569587

**Published:** 2020-10-22

**Authors:** Liang Deng, Yuwei Han, Chi Tang, Qingchao Liao, Zheng Li

**Affiliations:** Department of Animal Genetics, Breeding and Reproduction, College of Animal Science and Veterinary Medicine, Shenyang Agricultural University, Shenyang, China

**Keywords:** proteomics, jennies, early pregnancy, biomarkers, serum proteins

## Abstract

Early pregnancy in jennies is routinely determined by palpation per rectum or ultrasonography and also by detecting steroid hormone and chorionic gonadotropin levels in the blood, plasma, and serum. Herein we applied label-free mass spectrometry-based quantitative proteomics to identify serum proteins that were differentially expressed between early pregnant (day 45 after ovulation) and non-pregnant jennies. Bioinformatics analysis allowed illustration of pathways potentially involved in early pregnancy. We identified 295 proteins from a total of 2,569 peptides. Twenty-five proteins (22 upregulated and three downregulated) were significantly differentially expressed between the early pregnant and non-pregnant groups. The majority of the differentially expressed proteins were involved in defense response, early embryonic development, and hormone signaling pathways. Furthermore, functional protein analyses suggested that proteins were involved in binding, enzyme inhibitor activity, and enzyme regulator activity. Five serum proteins—granulin precursor/acrogranin, transgelin-2, fibronectin, fibrinogen-like 1, and thrombospondin 1—can be considered as novel, reliable candidates to detect pregnancy in jennies. To the best of our knowledge, this is the first study to use label-free mass spectrometry-based quantitative proteomics to analyze serum proteins during early pregnancy in jennies. Our results should facilitate the identification of valuable pregnancy diagnostic markers in early pregnant jennies.

## Introduction

Donkey (*Equus asinus*), a member of the equids, provides substantial societal benefits to humans (1). Considering its novel and evolving role in milk, meat, and skin production and in animal-assisted therapy, there has been a renewed interest in this species. A decline in donkey population and genetic diversity has become a global trend; thus, successful pregnancies are essential for maintaining effective population size and genetic diversity (2). Pregnancy in jennies (female donkeys) can be determined by palpation per rectum at around 40 days after ovulation (3) or by ultrasonography at around 10 days after ovulation (4). Steroid hormone variations in the blood of pregnant jennies have been previously investigated. Detecting progesterone and estradiol concentrations in the blood, plasma, and serum has become a method for pregnancy diagnosis in jennies (5).

There is a relatively high concentration of proteins present in the blood, plasma, and serum. The serum concentration of proteins in donkeys is 58–82 g/L (6). Molecular events are regulated by the expression of proteins in the blood. Thus, blood proteome could be used to understand biological mechanisms at a molecular level. The blood, plasma, and serum are widely utilized for diagnostic purposes in clinical practice (7) and for the discovery of novel biomarkers (8).

With the development of proteomics, different fractions of the blood proteome can now be analyzed in detail. Thus far, several proteomics approaches have been widely and effectively applied to explore the blood proteome of equids. Two-dimensional (2D) gel electrophoresis is routinely used for assessing quantitative changes in serum and/or plasma proteins of horses, such as for establishing the serum protein map (9, 10), evaluating changes in plasma proteins after prolonged physical exercise (11), and comparing differences in serum protein expression profiles between distinct breeds (12). Henze et al. (13) evaluated genetic differences in the serum proteome of horses, donkeys, and mules using surface-enhanced laser desorption–ionization time-of-flight mass spectrometry (MS), and they found considerable differences in the proteome of horses and donkeys and that the proteome of mules showed a higher similarity to donkeys than to that of horses.

It is well-known that there is an increase in blood protein synthesis during pregnancy (14). In several species, factors that help diagnose pregnancy have been subjected to in-depth research using proteomics. Some previous studies used high-resolution 2D gel electrophoresis in bovine (15, 16) and buffaloes (17) to identify potential pregnancy-specific markers in the serum and plasma. Furthermore, in pregnant women who delivered at term, Scholl et al. (18) used two-dimensional difference in-gel electrophoresis as well as isotope tagging for relative and absolute quantitation to assess changes in relative protein abundance between paired serum samples collected in the first and third trimesters.

A remarkable feature of equine pregnancy is the development of the invasive trophoblast of the chorionic girdle and its formation of gonadotrophin-secreting endometrial cup cells in early pregnancy. Equine chorionic gonadotropin, the main secretory product of the endometrial cups, is secreted during days 40–150 of pregnancy (19). During this period, numerous proteins are involved in the process of fetomaternal interactions. To the best of our knowledge, there are no published data on serum proteins during early pregnancy in jennies. Thus, using label-free MS-based quantitative proteomics, we herein aimed to identify specific serum proteins during early pregnancy in jennies.

## Materials and Methods

### Animals and Sampling

We used Liaoxi donkeys in this study (20). Nine pregnant jennies at day 45 after ovulation and nine non-pregnant jennies (aged 4–8 years) were randomly chosen at a breeding farm in Liaoning Province, China. The animals were considered healthy based on veterinarian records, physical examination, and reproductive tract examination performed by transrectal palpation and ultrasonography. A “B mode” ultrasound scanner equipped with a 7.0 MHz linear array multi-frequency transducer (WED-3000, Shenzhen WELLD, China) was used to monitor ovarian and uterine activity during estrus and pregnancy. The presence of a preovulatory follicle (≥3.5 cm) indicated impending ovulation. Artificial insemination was performed on the day of ovulation, using fresh semen from the same fertile jack donkey. The animals were first examined for pregnancy 12–15 days after ovulation, and re-examined at 40 and 45 days after ovulation to confirm the pregnancy status. Nine jennies that continued pregnancy to 45 days after ovulation were included into the “pregnant group” (P group). Further, nine jennies that repeated their cyclic estrous activity 21–22 days after the last estrus were included into the “non-pregnant group” (NP group). All jennies were maintained in a stable and outdoor paddock with *ad libitum* access to a mixture of cereal straw and grass hay, maize, bran, peas, minerals, vitamins, and water.

Blood samples from the P and NP groups were collected at day 45 after ovulation, and obtained immediately after confirming the non-pregnant state, respectively. Blood samples were collected at 9–10 am. The blood was drawn from the jugular vein and dispensed into 10-mL disposable vacuum tubes (BD Vacutainer, USA) without an anticoagulant. Within 1 h of collection, the samples were centrifuged at 3,000 *g* for 10 min at 4°C, and the serum thus obtained was stored at −80°C until needed. This study was conducted with the approval of Shenyang Agricultural University Animal Care and Use Committee (Permit no.: 201906025).

### Protein Extraction and Digestion

To avoid the influence of individual differences on serum proteins, the nine serum samples obtained from each group were randomly divided into three subgroups. The three serum samples (10 μL/sample) within each subgroup were equally pooled to obtain three biological replicates from each group. Proteins were extracted using SDT lysis buffer (4% SDS, 100 mM DTT, 100 mM Tris-HCl, pH 8.0), and protein concentration was determined using the BCA protein assay kit (Bio-Rad, USA). The protein samples were digested with trypsin, in accordance with the filter-aided sample preparation method (21). The digested peptides of each sample were desalted on C18 cartridges [Empore™ SPE C18 Cartridges (standard density), 7 mm bed I.D., 3 mL volume, Sigma], concentrated by vacuum centrifugation, and reconstituted in 40 μL of 0.1% (v/v) formic acid.

### Liquid Chromatography (LC)–Electrospray Ionization (ESI)–Tandem MS (MS/MS)

LC–ESI–MS/MS was performed on a Q-Exactive Plus mass spectrometer coupled with an EASY 1200 nano-LC System (Thermo Fisher Scientific, Bremen, Germany). LC–ESI–MS/MS settings were the same as those stated in a previous study (22). Briefly, 3 μg of the peptide mixture was first loaded onto a trap column (100 μm inner diameter, 20 mm long, 5 μm resin, C18, Dr. Maisch GmbH, Ammerbuch, Germany) in buffer A (0.1% formic acid in water). Reversed-phase high-performance LC was then performed with the EASY 1200 nano LC System (Thermo Fisher Scientific, Bremen, Germany) using a self-packed column (75 μm inner diameter, 150 mm long, 3 μm ReproSil-Pur C18 beads, 120 Å, Dr. Maisch GmbH, Ammerbuch, Germany), and the peptide mixture was separated with a linear gradient of buffer B (0.1% formic acid in 85% acetonitrile) at a flow rate of 300 nL/min controlled by IntelliFlow for 120 min. MS/MS data were acquired using a data-dependent top 20 method by dynamically choosing the most abundant precursor ions. The survey scans were selected with an isolation window of 1.6 Thomson and fragmented by higher energy collisional dissociation with a normalized collision energy of 27 eV. The maximum ion injection times for the survey and MS/MS scans were 50 ms, and the ion target values were set to 1e6 and 1e5, respectively. Selected sequenced ions were dynamically excluded for 60 s.

### Sequence Database Searching and Protein Quantification

Raw MS data were analyzed using MaxQuant v1.6.0.16 and searched against UniProtKB *Equus* (28,987 total entries, downloaded on 15/09/2019 from http://www.uniprot.org). The initial search peptide tolerance and mass tolerance were set at 20 ppm for fragment ions. The main search peptide tolerance was 4.5 ppm. The search was performed based on an enzymatic cleavage rule of trypsin/P, and a maximum of two missed cleavage sites were allowed (21). The carbamidomethylation of cysteine residues was defined as a fixed modification, while protein N-terminal acetylation and methionine oxidation were specified as variable modifications for database searching. The false discovery rate for both peptide and protein identification was set to be <0.01 (23). Protein identification was supported by at least one unique peptide identification.

### Statistical and Bioinformatics Analysis

Differential proteins were analyzed for significant up- or downregulation, which was assessed using the R statistical computing software (v 3.2.1). Protein abundance information was collected to have at least two valid expression values in each group. Normality was assessed using the Shapiro–Wilk test. This exploratory analysis of the dataset showed that the variables were normally distributed; thus, the independent samples *t-*test was used to determine statistical significance for comparison between the P and NP groups. The significantly differentially expressed proteins (DEPs) were further inspected and the ones with a differential expression ratio of ≥1.5-fold or ≤0.66-fold (*P* < 0.05) were retained.

Analyses of bioinformatics data were performed with Perseus (24), Microsoft Excel, and R statistical computing software. The protein sequences of selected DEPs were extracted from UniProtKB/Swiss-Prot (25), gene ontology (GO) terms, and Kyoto Encyclopedia of Genes and Genomes (KEGG) database (http://geneontology.org/). GO and KEGG enrichment analyses were applied on the basis of Fisher's exact test in which whole quantified protein annotations were considered as background dataset, and only functional categories and pathways with *P* < 0.05 were recognized as significant. The protein–protein interaction (PPI) information of DEPs was retrieved using the STRING (http://string-db.org/) database and Cytoscape software (26).

## Results

### Identification and Quantification of the Serum Proteome

**Figure 1:**
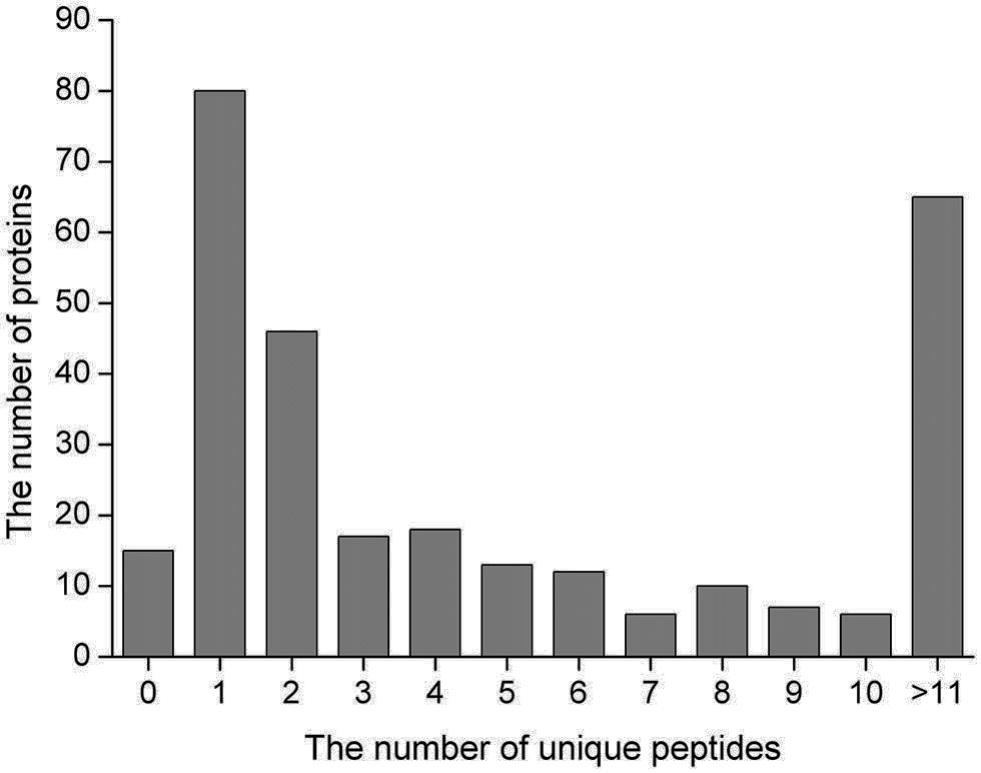
Unique peptides for the identified proteins.

We herein used the label-free MS-based quantitative proteomics to investigate serum proteins in jennies, which led to the identification of 295 proteins (Supplementary Table 1) in the serum of early pregnant and non-pregnant jennies with a high confidence level (≥1 unique peptides with false discovery rate of <1%). Overall, 275 proteins were common between the P and NP groups. Of the 295 proteins that were quantified, 27.1% (80/295) were inferred from one peptide, and 22.0% (65/295) were proteins with >11 unique peptides (Figure 1). The molecular weight of the identified proteins ranged from 1.53 to 881.23 kDa. Albumin, α-2-macroglobulin, serotransferrin, apolipoprotein A1, and haptoglobin were the major serum proteins. Detailed information pertaining to the identified 2,569 peptides is shown in Supplementary Table 2.

### Differentially Expressed Serum Protein Profiles

**Figure 2:**
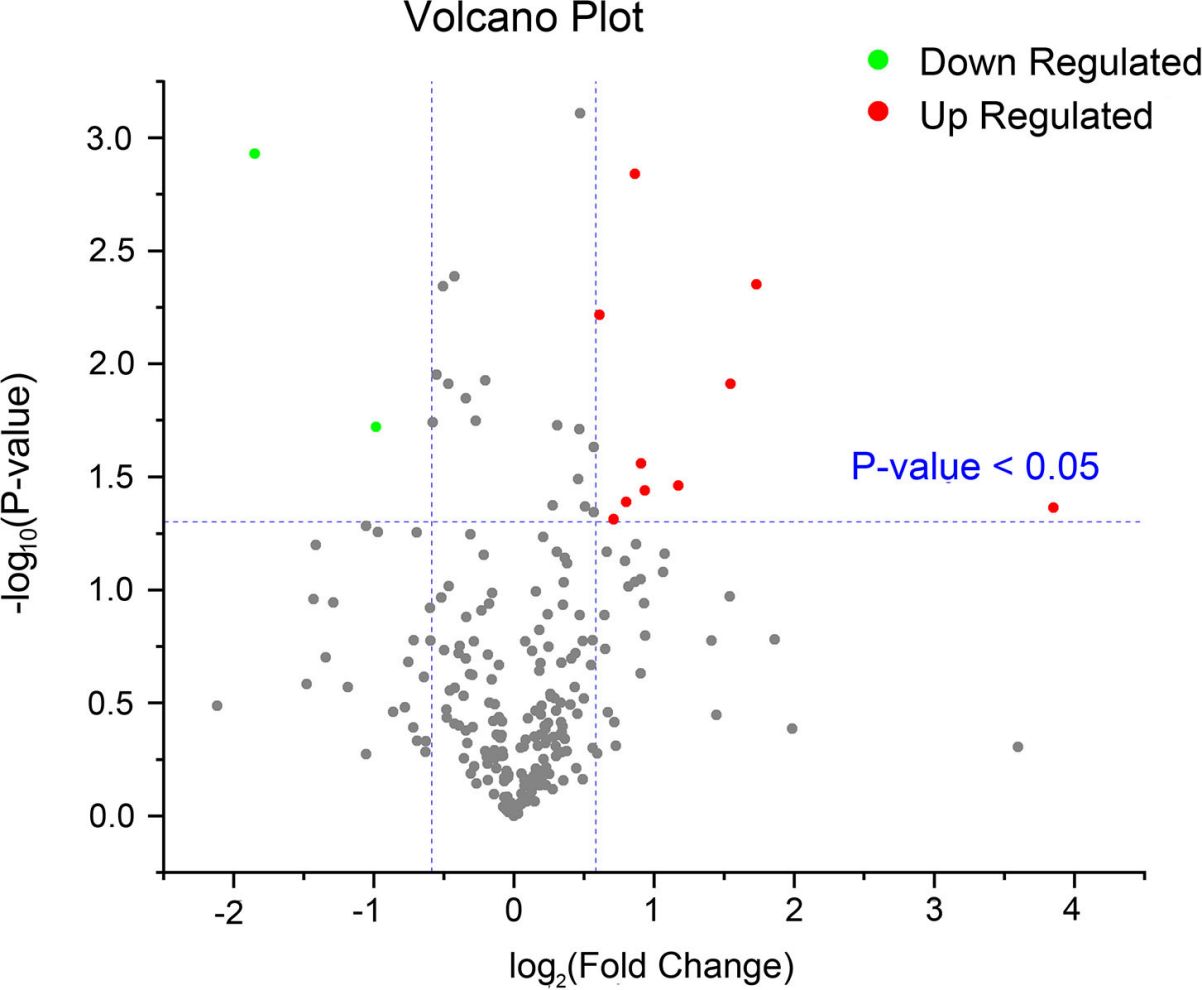
A volcano plot of common proteins identified in the serum of early pregnant and non-pregnant jennies. This plot was drawn on the basis of the fold change and *P*-value between the P and NP groups. Red and green dots represent upregulated and downregulated common proteins, respectively (fold change ≥1.5 and *P* < 0.05). Gray dots represent proteins without any difference.

Twenty-five proteins (22 upregulated and three downregulated) were found to be significantly differentially expressed between the P and NP groups (≥1.5-fold, Student's *t-*test, *P* < 0.05) (Table 1, Supplementary Table 3). Among the 25 DEPs, 12 (10 upregulated and two downregulated) were common between the P and NP groups. According to the fold change and *P*-value between the groups, a volcano plot was drawn to illustrate significant differences (Figure 2). In addition, among the 25 DEPs, there were 12 and one proteins that were uniquely expressed in the P and NP groups, respectively.

**Table 1:** Differentially expressed proteins in the serum of early pregnant (P group) and non-pregnant (NP group) jennies.

**UniProt accession**	**Protein names**	**Gene names**	**Coverage (%)^b^**	**Molecular weight (kDa)**	**FC^a^**	***P*-value**	**Change^e^**
F6YR34	Thrombospondin 1	THBS1	21.1	129.56	14.43	0.0432	↑
F7CN05	Fibronectin	FN1	39.7	252.55	3.32	0.0045	↑
F6RMK1	C-X-C motif chemokine	LOC100630489	31.5	11.835	2.92	0.0122	↑
F6ZRF6	Serpin family A member 7	SERPINA7	18.6	46.64	2.25	0.0346	↑
A0A0A1E971	Immunoglobulin lambda light chain variable region (Fragment)	IGL	30.4	23.267	1.91	0.0363	↑
F7ATS5	Golgi membrane protein 1	GOLM1	12.5	41.218	1.87	0.0276	↑
Q5IF07	Insulin-like growth factor binding protein-2 (Fragment)	IGFBP-2	17.7	16.042	1.82	0.0014	↑
F6WA57	Lymphocyte cytosolic protein 1	LCP1	35.8	70.347	1.74	0.0409	↑
F6Y950	Chromosome 2 C4orf33 homolog	C2H4orf33	6.5	23.394	1.64	0.0486	↑
F6Y2H3	Peptidase D	PEPD	12.2	54.827	1.53	0.0061	↑
A0A0A1E3W4	Immunoglobulin lambda light chain variable region (Fragment)	IGL	25.4	23.511	P^c^		↑
F6VP61	Serpin family B member 10	SERPINB10	5.8	45.412	P^c^		↑
F6QXN5	Transgelin2	TAGLN2	9.3	23	P^c^		↑
F6RCZ8	Triggering receptor expressed on myeloid cells like 1	TREML1	5	33.597	P^c^		↑
F6RLT8	Calpain small subunit 1	CAPNS1	4.9	28.099	P^c^		↑
F6RMQ1	GLI pathogenesis related 2	GLIPR2	17.2	16.905	P^c^		↑
F6T962	EGF containing fibulin extracellular matrix protein 2	EFEMP2	6.5	49.439	P^c^		↑
F6ZEJ2	HGF activator	HGFAC	7.2	55.76	P^c^		↑
F7AB03	Granulin precursor	GRN	6.6	63.195	P^c^		↑
F7DIN1	Fibrinogen like 1	FGL1	4.4	36.475	P^c^		↑
F7E2K1	Uncharacterized protein	N/A	2.1	72.262	P^c^		↑
P19794	Lutropin/choriogonadotropin subunit beta	LHB	10.1	17.943	P^c^		↑
F7BSN5	Uncharacterized protein	N/A	7.6	59.264	0.51	0.0190	↓
F7DU87	Uncharacterized protein	BPIFA2	39.8	26.915	0.28	0.0012	↓
F7ATL5	Keratin 14	KRT14	8.2	41.036	NP^d^		↓

a *FC, fold change, mean value of peak area obtained from the P group/mean value of peak area obtained from the NP group. If the fold-change value was >1.5, the relative content of serum proteins in the P group was higher that than in the NP group, and if the fold-change value was <0.67, the relative content of serum proteins in the P group was less than that in the NP group*.

b *coverage (%) = percentage of the protein sequence covered by identified peptides*.

c *uniquely expressed serum proteins in the P group*.

d *uniquely expressed serum proteins in the NP group*.

e *“↑” = upregulated serum proteins; “↓” = downregulated serum proteins*.

### Bioinformatics Analysis of DEPs

**Figure 3:**
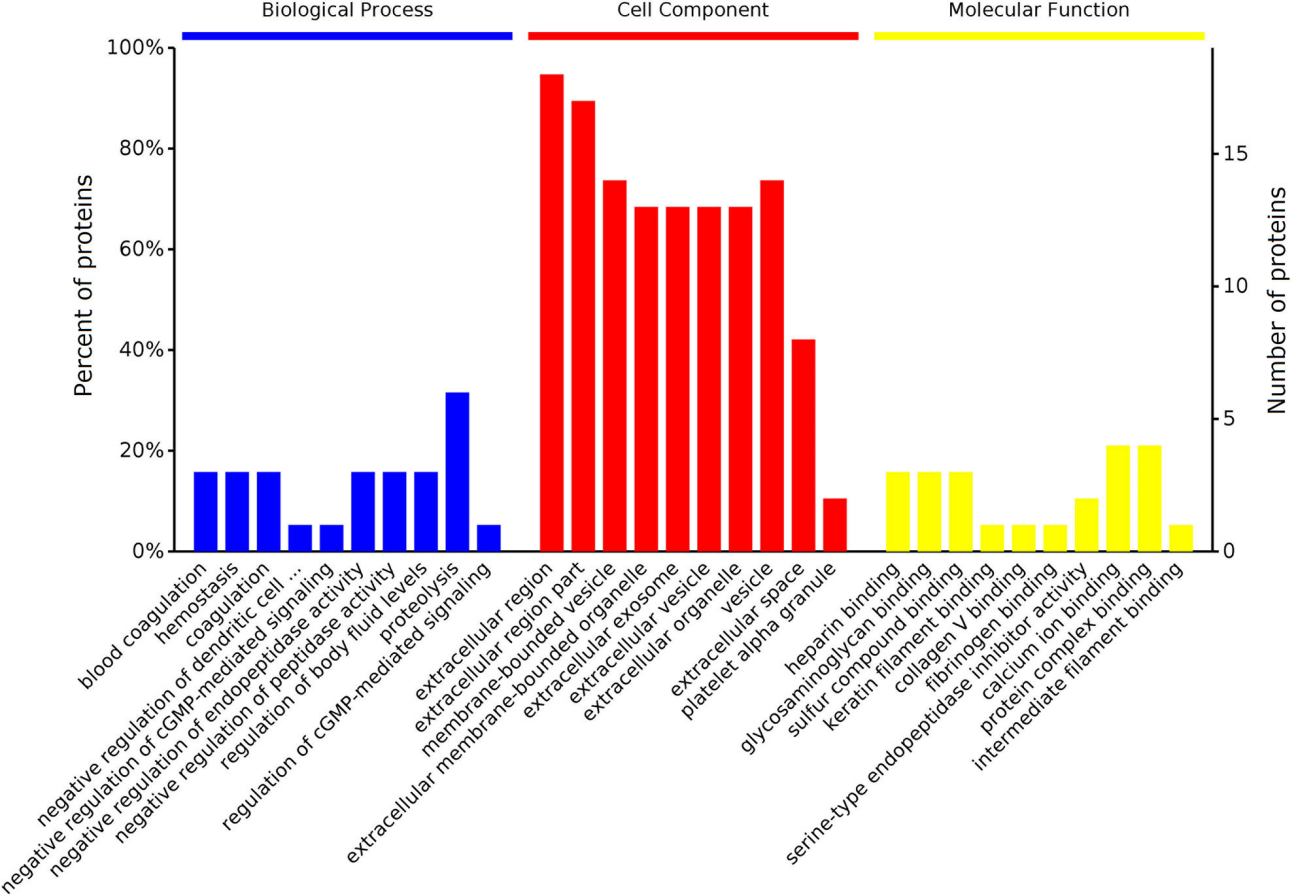
GO enrichment analyses of DEPs identified in the serum of early pregnant and non-pregnant jennies. Most proteins could be divided into three major categories: biological process, cellular component, and molecular function.

The 25 DEPs were classified into three distinctive functional sets through GO enrichment analysis, namely biological process (BP), cellular component (CC), and molecular function (MF) (Figure 3), with a corrected statistically significant level (*P* < 0.05) based on Fisher's exact test. The most prevalent BPs were response to stimulus, regulation of cellular process, protein metabolic process, and proteolysis. The most enriched CCs were extracellular region, vesicle, extracellular organelle, and extracellular space, and in the MF category, DEPs were mainly involved in binding, enzyme inhibitor activity, and enzyme regulator activity.

**Figure 4:**
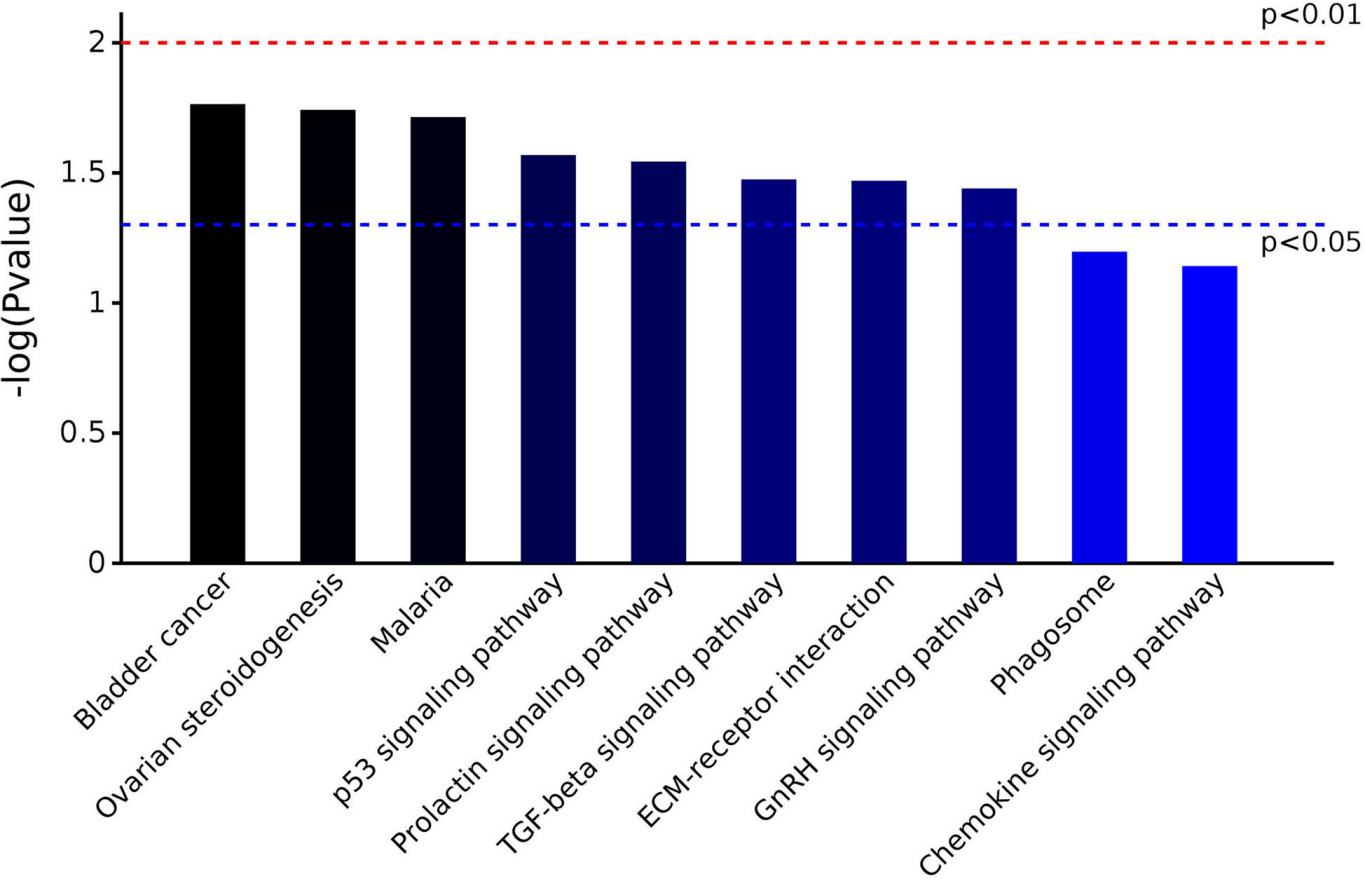
KEGG pathway enrichment analysis of DEPs identified in the serum of early pregnant and non-pregnant jennies.

The 25 DEPs were related to 17 KEGG pathways; the first 10 pathways are shown in Figure 4. The prevalent pathways related to DEPs were involved in bladder cancer, ovarian steroidogenesis, malaria, p53 signaling, prolactin signaling, TGF-beta signaling, and GnRH signaling. Detailed information on the functional enrichment of DEPs is present in Supplementary Table 4.

### PPI Analysis

**Figure 5:**
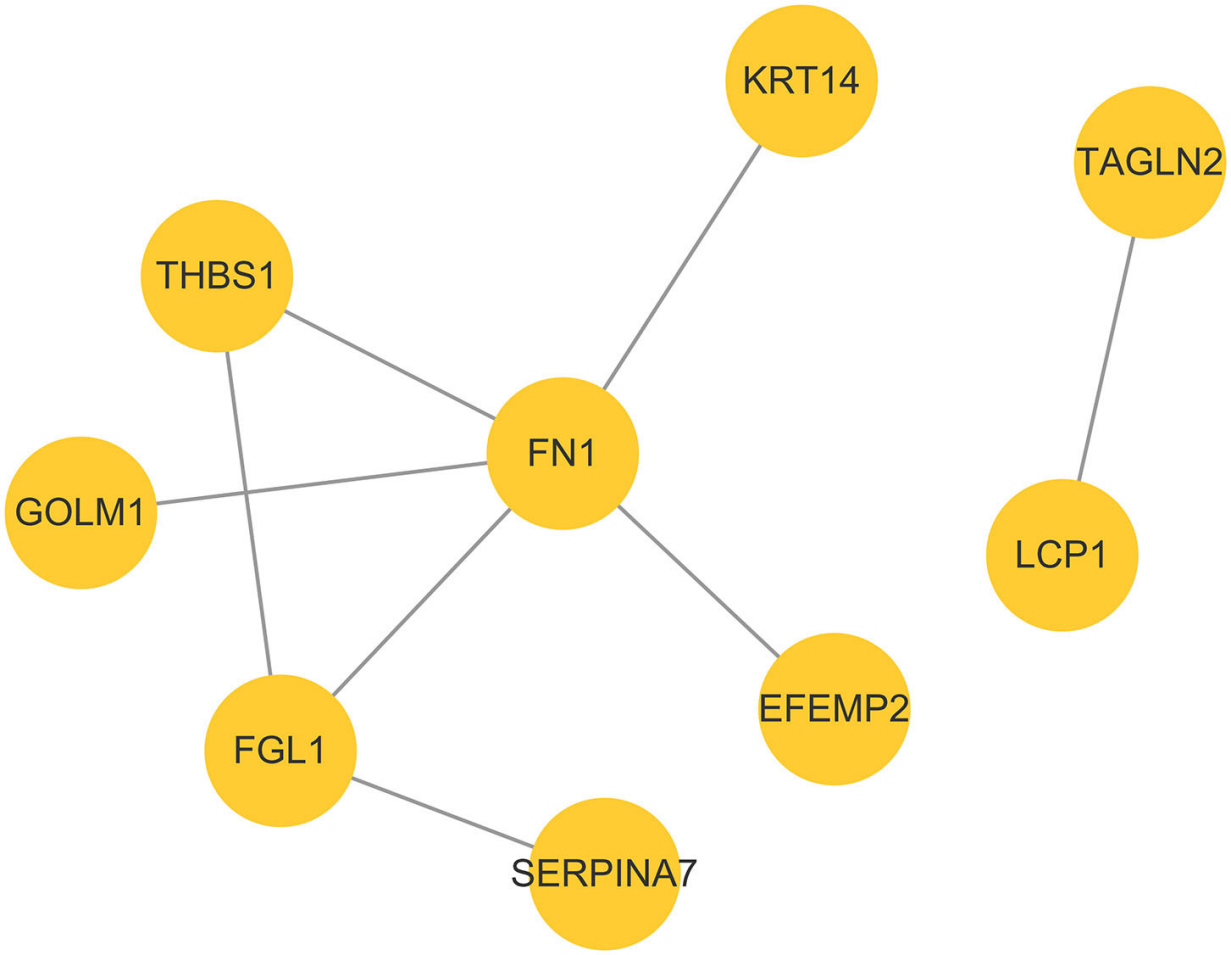
Protein–protein interaction network analysis based on DEPs in the P group vs. the NP group.

Color-coded networks were generated to represent different types of evidence for the biological crosslink between DEPs. The networks contained nine DEPs, and the core factors were fibronectin (FN1), fibrinogen-like 1 (FGL1), and thrombospondin 1 (THBS1). These three proteins were connected by 2–5 nodes (Figure 5).

## Discussion

Serum proteins during early pregnancy in jennies have not been previously investigated. In this study, we generated proteome profiles of the serum obtained from jennies using label-free MS-based quantitative proteomics, which led to the identification of 25 DEPs. This is the first comprehensive study to explore the potential biological significance of DEPs between early pregnant and non-pregnant jennies, providing valuable insights into pregnancy-specific serum proteins that could be applied for developing pregnancy diagnostic markers in early pregnant jennies.

The label-free MS-based quantitative proteomics method, i.e., spectral counting, compares the number of mass spectra assigned to each protein (27). It offers several advantages such as high sensitivity, real-time measurements, rapid and multiplexed detection, larger quantification dynamic range, and improved reproducibility (28), as compared with the low identification rate of the 2D gel electrophoresis method (27). The label-free MS-based quantitative proteomics method has thus emerged as a promising technique for quantitative protein profiling of complex biological samples, such as serum (29).

Several proteomics-based studies involving various animal species have reported the identification of potential pregnancy-specific serum proteins. These studies have mostly used high-resolution 2D gel electrophoresis and MS for this purpose (15–17). Four differentially expressed serum proteins, namely transferrin, albumin, IgG2a heavy chain constant region, and immunoglobulin gamma heavy chain variable region, were identified as pregnancy-specific proteins in Holstein dairy cattle by 2D gel electrophoresis plus matrix-assisted laser desorption/ionization–time of flight (MALDI–TOF) MS (15). In another study, 2D fluorescence difference gel electrophoresis plus MALDI-TOF MS were used to analyze serum proteins obtained from Holstein dairy cattle, which led to the identification of 16 DEPs spots; among them, upregulated proteins, such as conglutinin precursor, modified bovine fibrinogen, and IgG1, and downregulated proteins, such as hemoglobin, complement component 3, bovine fibrinogen, and IgG2a heavy chain constant region, were considered as pregnancy-specific proteins (16). Further, upon exploration of serum pregnancy-specific proteins in Murrah buffaloes, synaptojanin-1, apolipoprotein a-1, apolipoprotein b, keratin 10, and von Willebrand factors were identified by 2D gel electrophoresis plus MALDI-TOF MS (17). In this study, none of the DEPs identified by us using label-free MS-based quantitative proteomics have been reported in previous studies, indicating interspecific differences.

So far, some pregnancy-specific proteins, such as pregnancy-specific glycoproteins, have been found in trophoblast cells of pregnant mares (30); however, to the best of our knowledge, the identification of pregnancy-specific serum proteins based on proteomics has not been performed in horses. Further studies need to be conducted to reveal more biomarkers of early pregnancy and to compare differences among different *Equus* species.

Herein many DEPs, such as FN1, FGL1, THBS1, granulin precursor/acrogranin (GRN), C-X-C motif chemokine, triggering receptor expressed on myeloid cells-1 (TREML1), and keratin 14 (KRT14), were identified in the serum obtained from early pregnant jennies, and these were mainly associated with defense response. FN1 usually exists as a dimeric glycoprotein and is a ubiquitous and abundant soluble constituent of the plasma and other body fluids; it is also a part of the insoluble extracellular matrix (31). This extracellular fibronectin niche plays essential roles in wound healing, malignant transformation, inflammation, infection, hemostasis, and thrombosis (32). FGL1 belongs to the fibrinogen family and is primarily secreted from hepatocytes. It is involved in the process of blood clotting (33), and a study reported that recombinant human IL-6 could induce FGL1 expression in Hep G2 cells and that serum FGL1 levels were also enhanced after induction of acute inflammation in rats by subcutaneous injection of turpentine oil (34). THBS1 interacts with a cohort of target molecules through its multifunctional domains and participates in normal development and homeostasis maintenance (35). It also plays an important role in the regulation of coagulation, antiangiogenesis, wound healing, and immune response (36). The 6-kDa form of GRN has been invoked in wound repair (37). GRN is also a potent inhibitor of the inflammatory cytokine tumor necrosis factor-α and regulate inflammation (38). Both C-X-C motif chemokine and TREML1 are involved in platelet activation. KRT14, encoding a basal cell keratin, is expressed during the wound healing process and participates in cell morphogenesis and epidermis development (39). Changes in the serum level of these proteins indicates the involvement of a regulatory mechanism of defense response during early pregnancy.

The presence of many pregnancy-specific proteins associated with early embryonic development have been reported in the serum, other body fluids, and tissues of animals. We found that the expression of GRN and transgelin-2 (TAGLN2) was unique. GRN is an embryo-derived growth factor, which possesses growth regulatory activities principally toward epithelial cells. GRN has been reported to regulate the appearance of the epithelium in developing mouse blastocysts, growth of the trophectoderm, and/or function of the embryonic epithelium (40). As a member of the transgelin family, TAGLN2 plays a significant role in the regulation of F-actin remodeling, which is important for trophoblast cell adhesion and invasion. TAGLN2 may be a potential target to improve embryo implantation rates in *in vitro* fertilization (41). Furthermore, the significantly upregulated proteins FN1, FGL1, and THBS1 are also seemingly involved in the establishment and maintenance of pregnancy. FN1 plays major roles in cell adhesion, migration, growth, and differentiation. It also binds to a number of biologically important molecules (42). Moreover, it is indispensable for embryogenesis; George et al. (43) reported that FN1-deficient mice died around embryonic day 10.5 due to severe defects in mesoderm. FGL1 is also expressed in the placenta and increases the proliferation of trophoblasts through an ERK-dependent pathway (44). THBS1 is reportedly involved in the organization of the cytoskeleton and in the process of adhesion (45). It has also been reported to participate in the adhesiveness of the embryo during the peri-implantation period in pigs (46). Considering our findings pertaining to the differential expression of GRN, TAGLN2, FN1, FGL1, and THBS1 in the serum of early pregnant jennies, which have been proved to play an important role in early embryonic development in other species, these five proteins seem to be novel, reliable candidates to detect pregnancy in jennies.

Equine chorionic gonadotropin is a pregnancy-specific glycoprotein hormone produced by the placenta between day 40 and 150 of pregnancy, and it shows FSH- and LH-like biological activities (19). Equids possess a single lutropin/choriogonadotropin subunit-β (LHB) gene, which confers specificity on the intact hormone (47). In our study, LHB was noted to be uniquely expressed in the serum of pregnant jennies and was probably involved in ovarian steroidogenesis and prolactin signaling and GnRH signaling pathways, according to the KEGG pathway-based enrichment analysis. LHB has been identified as a marker of pregnancy in jennies, although it shows considerably lower FSH-like activity than horse LHB (47).

## Conclusion

To summarize, this is the first study to report a comprehensive analysis of DEPs in the serum of early pregnant and non-pregnant jennies using label-free MS-based quantitative proteomics. Twenty-two upregulated and three downregulated proteins were significantly differentially expressed in the P group, and these were mainly associated with the defense response, early embryonic development, and hormone signaling pathways. The differences in the expression profiles helped identify candidate proteins to explore pregnancy diagnostic markers related to early pregnancy in jennies. Five serum proteins—GRN, TAGLN2, FN1, FGL1, and THBS1—appear to be novel, reliable candidates to detect pregnancy in jennies. Further studies should be conducted to elucidate their functions in jennies.

## Data Availability Statement

The datasets presented in this study can be found in online repositories. The names of the repository/repositories and accession number(s) can be found in the article/Supplementary Material.

## Ethics Statement

The animal study was reviewed and approved by Shenyang Agricultural University Animal Care and Use Committee.

## Author Contributions

LD performed the experiments. YH and CT participated in data analysis. CT, QL, and ZL helped with sampling. LD and YH edited the manuscript. All authors read and approved the final manuscript.

## Conflict of Interest

The authors declare that the research was conducted in the absence of any commercial or financial relationships that could be construed as a potential conflict of interest.

## References

[1] Ali M Baber M Hussain T Awan F Nadeem A. The contribution of donkeys to human health. Equine Vet J. (2014) 46:766–7. 10.1111/evj.12337 25319161

[2] Tibary A Sghiri A Bakkoury M Reproduction. In: Duncan J, Hadrill D, editors. The Professional Handbook of the Donkey. 4th ed. London: Whittet Books (2008). p. 314–41.

[3] The Donkey Sanctuary Reproductive system. In: Evans L, Crane M, editors. The Clinical Companion of the Donkey. Leicester: Troubador (2018). p. 67–79.

[4] Meira C Ferreira JC Papa FO Henry M. Ultrasonographic evaluation of the conceptus from days 10 to 60 of pregnancy in jennies. Theriogenology. (1998) 49:1475–82. 10.1016/S0093-691X(98)00093-4 10732011

[5] Meira C Ferreira JC Papa FO Henry M. Ovarian activity and plasma concentrations of progesterone and estradiol during pregnancy in jennies. Theriogenology. (1998) 49:1465–73. 10.1016/S0093-691X(98)00092-2 10732010

[6] Svendsen MBE Biochemical parameters. In: Duncan J, Hadrill D, editors. The Professional Handbook of the Donkey. 4th ed. London: Whittet Books (2008). p. 381.

[7] Ve′gva′ri Á Welinder C Lindberg H Fehniger TE Marko-Varga G. Biobank resources for future patient care: developments, principles and concepts. J Clin Bioinforma. (2011) 1:24. 10.1186/2043-9113-1-24 21923917 PMC3197484

[8] Beck HC Overgaard M Rasmussen LM Plasma proteomics to identify biomarkers – application to cardiovascular diseases. Transl Proteomics. (2015) 7:40–8. 10.1016/j.trprot.2015.01.001

[9] Miller I Friendlein A Tsangaris G Maris A Fountoulakis M Gemeiner M. The serum proteome of *Equus caballus*. Proteomics. (2004) 4:3227–34. 10.1002/pmic.200400846 15378688

[10] Lepczyński A Ozgo M Dratwa-Chałupnik A Robak P Pyć A Zaborski D. An update on medium-and low-abundant blood plasma proteome of horse. Animal. (2018) 12:76–87. 10.1017/S1751731117001409 28689516

[11] Scoppetta F Tartagila M Renzone G Avellini L Gaiti A Scaloni A. Plasma protein changes in horse after prolonged physical exercise: a proteomic study. J Proteomics. (2012) 75:4494–504. 10.1016/j.jprot.2012.04.014 22546489

[12] Barsuren E Namkhai B Kong HS. Differences in serum protein 2D gel electrophoresis patterns of Przewalski's (Mongolian wild horse) and thoroughbred horses. Anim Sci J. (2015) 86:443–8. 10.1111/asj.12303 25533201

[13] Henze A Aumer F Grabner A Raila J Schweigert FJ. Genetic differences in the serum proteome of horses, donkeys and mules are detectable by protein profiling. Brit J Nutr. (2011) 106:S170–3. 10.1017/S0007114511000845 22005420

[14] Larsson A Palm M. Hansson LO Axelsson O. Reference values for clinical chemistry test during normal pregnancy. BJOG Int J Obstet Gynaecol. (2008) 115:874–81. 10.1111/j.1471-0528.2008.01709.x 18485166

[15] Jin DI Lee HR Kim HR Lee HJ Yoon JT Park CS 151 Proteomics analysis of pregnancy-specific serum proteins in bovine. Reprod Fertil Dev. (2005) 18:183 10.1071/RDv18n2Ab151

[16] Lee JE Lee JY Kim HR Shin HY Lin T Jin DI. Proteomic analysis of bovine pregnancy-specific serum proteins by 2D fluorescence difference gel electrophoresis. Asian Australas J Anim Sci. (2015) 28:788–95. 10.5713/ajas.14.0790 25925056 PMC4412975

[17] Balhara AK Gupta M Mohanty AK Phulia SK Sharma RK Singh S Changes in sera proteome in relation to day of pregnancy in early pregnant buffaloes. Indian J Anim Sci. (2014) 84:400–9. 10.2478/aoas-2014-0018

[18] Scholl PF Cole RN Ruczinski I Gucek M Diez R Rennie A. Maternal serum proteome changes between the first and third trimester of pregnancy in rural Southern Nepal. Placenta. (2012) 33:424–32. 10.1016/j.placenta.2012.02.009 22385826 PMC4703092

[19] Antczak DF de Mestre AM Wilsher S Allen WR. The equine endometrial cup reaction: a fetomaternal signal of significance. Annu Rev Anim Biosci. (2013) 1:419–42. 10.1146/annurev-animal-031412-103703 25387026 PMC4641323

[20] Deng L Shi S Li J Tang C Liao Q Xie P. A cross-sectional survey of foaling-related parameters of Jennies (*Equus asinus*) under smallholder farm conditions in Northeast China. J Equine Vet Sci. (2020) 87:102928. 10.1016/j.jevs.2020.102928 32172918

[21] Wiśniewski JR Zougman A Nagaraj N Mann M. Universal sample preparation method for proteome analysis. Nat Methods. (2009) 6:359–62. 10.1038/nmeth.1322 19377485

[22] Cao X Song D Yang M Yang N Ye Q Tao D. Comparative analysis of whey N-glycoproteins in human colostrum and mature milk using quantitative glycoproteomics. J Agric Food Chem. (2017) 65:10360–7. 10.1021/acs.jafc.7b04381 29110469

[23] Luber CA Cox J Lauterbach H Fancke B Selbach M Tschopp J. Quantitative proteomics reveals subset-specific viral recognition in dendritic cells. Immunity. (2010) 32:279–89. 10.1016/j.immuni.2010.01.013 20171123

[24] Tyanova S Temu T Sinitcyn P Carlson A Hein MY Geiger T. The Perseus computational platform for comprehensive analysis of (prote)omics data. Nat Methods. (2016) 13:731–40. 10.1038/nmeth.3901 27348712

[25] Boutet E Lieberherr D Tognolli M Schneider M Bansal P Bridge AJ. UniProtKB/Swiss-Prot, the manually annotated section of the UniProt KnowledgeBase: How to use the entry view. Methods Mol Biol. (2016) 1374:23–54. 10.1007/978-1-4939-3167-5_2 26519399

[26] Shannon P Markiel A Ozier O Baliga NS Wang JT Ramage D. Cytoscape: a software environment for integrated models of biomolecular interaction networks. Genome Res. (2003) 13:2498–504. 10.1101/gr.1239303 14597658 PMC403769

[27] Zhu W Smith JW Huang C. Mass spectrometry-based label-free quantitative proteomics. J Biomed Biotechnol. (2010) 2010:840518. 10.1155/2010/840518 19911078 PMC2775274

[28] Ray S Reddy PJ Jain R Gollapalli K Moiyadi A Srivastava S. Proteomic technologies for the identification of disease biomarkers in serum: advances and challenges ahead. Proteomics. (2011) 11:2139–61. 10.1002/pmic.201000460 21548090

[29] Wang W Zhou H Lin H Roy S Shaler TA Hill LR Quantification of proteins and metabolites by mass spectrometry without isotopic labeling or spiked standards. Anal Chem. (2003) 75:4818–26. 10.1021/ac026468x 14674459

[30] Aleksic D Blaschke L Mißbach S Hänske J Weiß W Handler J. Convergent evolution of pregnancy-specific glycoproteins in human and horse. Reproduction. (2016) 152:171–84. 10.1530/REP-16-0236 27280409

[31] Wierzbicka-Patynowski I Schwarzbauer JE. The ins and outs of fibronectin matrix assembly. J Cell Sci. (2003) 116:3269–76. 10.1242/jcs.00670 12857786

[32] Ni H. Unveiling the new face of fibronectin in thrombosis and hemostasis. J Thromb Haemost. (2006) 4:940–2. 10.1111/j.1538-7836.2006.01899.x 16689738

[33] Rijken DC Dirkx SP Luider TM Leebeek FW. Hepatocyte-derived fibrinogen-related protein-1 is associated with the fibrin matrix of a plasma clot. Biochem Biophys Res Commun. (2006) 350:191–4. 10.1016/j.bbrc.2006.09.018 16996032

[34] Liu Z Ukomadu C. Fibrinogen-like protein 1, a hepatocyte derived protein is an acute phase reactant. Biochem Biophys Res Commun. (2008) 365:729–34. 10.1016/j.bbrc.2007.11.069 18039467 PMC2288651

[35] Lawler J Sunday M Thibert V Duquette M George EL Rayburn H. Thrombospondin-1 is required for normal murine pulmonary homeostasis and its absence causes pneumonia. J Clin Invest. (1998) 101:982–92. 10.1172/JCI1684 9486968 PMC508649

[36] Adams JC Lawler J The thrombospondins. Cold Spring Harb Perspect Biol. (2011) 3:a009712 10.1101/cshperspect.a009712 21875984 PMC3179333

[37] He Z Ong CH Halper J Bateman A. Progranulin is a mediator of the wound response. Nat Med. (2003) 9:225–9. 10.1038/nm816 12524533

[38] Kessenbrock K Fröhlich L Sixt M Lämmermann T Pfister H Bateman A. Proteinase 3 and neutrophil elastase enhance inflammation in mice by inactivating antiinflammatory progranulin. J Clin Invest. (2008) 118:2438–47. 10.1172/JCI34694 18568075 PMC2430496

[39] Founds SA Conley YP Lyons-Weiler JF Jeyabalan A Hogge WA Conrad KP. Altered global gene expression in first trimester placentas of women destined to develop preeclampsia. Placenta. (2009) 30:15–24. 10.1016/j.placenta.2008.09.015 19027158 PMC2667803

[40] Di'az-Cueto L Stein P Jacobs A Schultz RM Gerton GL. Modulation of mouse preimplantation embryo development by acrogranin (epithelin/granulin precursor). Dev Biol. (2000) 217:406–18. 10.1006/dbio.1999.9564 10625564

[41] Liang X Jin Y Wang H Meng X Tan Z. Huang T. Transgelin 2 is required for embryo implantation by promoting actin polymerization. FASEB J. (2019) 33:5667–75. 10.1096/fj.201802158RRR 30702937

[42] Pankov R Yamada KM. Fibronectin at a glance. J Cell Sci. (2002) 115:3861–3. 10.1242/jcs.00059 12244123

[43] George EL Georges-Labouesse EN Patel-King RS Rayburn H Hynes RO. Defects in mesoderm, neural tube and vascular development in mouse embryos lacking fibronectin. Development. (1993) 119:1079–91. 8306876 10.1242/dev.119.4.1079

[44] Kang L Li HY Ou HY Wu P Wang SH Chang CJ. Role of placental fibrinogen-like protein 1 in gestational diabetes. Transl Res. (2020) 218:73–80. 10.1016/j.trsl.2020.01.001 32006524

[45] Adams JC. Thrombospondins: multifunctional regulators of cell interactions. Annu Rev Cell Dev Biol. (2001) 17:25–51. 10.1146/annurev.cellbio.17.1.25 11687483

[46] Kolakowska J Souchelnytskyi S Saini RKR Franczak A. Proteomic analysis of the endometrium during early pregnancy in the domestic pig. Reprod Fertil Dev. (2017) 29:2255–68. 10.1071/RD16435 28416050

[47] Chopineau M Stewart F Allen WR. Cloning and analysis of the cDNA encoding the horse and donkey luteinizing hormone β-subunits. Gene. (1995) 160:253–6. 10.1016/0378-1119(95)00150-5 7642105

