# Supplementary material for: Label-Free Mass Spectrometry-Based Quantitative Proteomics Analysis of Serum Proteins During Early Pregnancy in Jennies (Equus asinus)
Source: Front Vet Sci. 2020 Oct 22;7:569587. doi: 10.3389/fvets.2020.569587 (PMC7642908; doi:10.3389/fvets.2020.569587)
Supplement: Supplementary file 5 [file Data_Sheet_1.docx]

Supplementary Material

# Supplementary Tables

**Supplementary Table 1.** Proteins identiﬁed in the serum of early pregnant and non-pregnant jennies with a high confidence level.

**Supplementary Table 2.** Detailed information pertaining to peptides identiﬁed in the serum of early pregnant and non-pregnant jennies.

**Supplementary Table 3.** Detailed information pertaining to differentially expressed proteins in the serum of early pregnant and non-pregnant jennies.

**Supplementary Table 4.** Detailed information on the functional enrichment of differentially expressed proteins in the serum of early pregnant and non-pregnant jennies.
